# Detection of airborne bovine coronavirus RNA in pen environments and exhaled breath of calves

**DOI:** 10.3168/jdsc.2026-1019

**Published:** 2026-03-12

**Authors:** Joanna Urbaniec, Lauren Brabiner, Peers Davies, Susan Gould, Joseph Neary

**Affiliations:** 1 Institute of Infection, Veterinary and Ecological Sciences, University of Liverpool, Liverpool, United Kingdom L69 7ZX; 2 Liverpool School of Tropical Medicine, Liverpool, United Kingdom L3 5QA

## Abstract

Bovine respiratory disease (BRD) is a significant economic and welfare concern in the cattle industry. Commingling (mixing) events create environments conducive to the spread of bovine coronavirus (BCV), a common calf BRD pathogen. Although BCV is thought to spread primarily through direct contact, the role of airborne transmission remains unclear. This study aimed to investigate the potential for airborne and fomite transmission of BCV among weaned (8–10 wk old) Angus × Holstein calves during a controlled commingling event. Eighty animals across 2 cohorts, raised under commercial farming conditions at 7 source farms, were enrolled in the study. Air, exhaled breath, and surface swabs were collected from solid-sided pens housing BCV-shedding calves and an adjacent, unoccupied separator pen. Exhaled breath was sampled using a novel device (IMADGENN) designed to capture particles at defined distances from the source. IMADGENN is a purpose-built chamber equipped with HEPA-filtered air supply and extraction systems to ensure low background aerosol levels and efficient sample collection. A low amount of BCV RNA was detected in the air of both occupied and unoccupied pens (at least 2.5 m away from the closest shedding animal), and surfaces in unoccupied pens were also found to be contaminated. Bovine coronavirus was also detected in exhaled breath of shedding calves, at a distance of at least 1.2 m from the sampled animal. In conclusion, our findings demonstrate that BCV particles are shed into the environment via exhaled breath, resulting in contamination of air and surfaces with viral RNA. These findings suggest that airborne dispersal and fomites may be a potential transmission route for virions and could contribute to BCV spread during commingling events.

## Highlights

• Bovine coronavirus RNA was detected in air in and around calf housing.
• Bovine coronavirus RNA was detected in exhaled breath at least 1.2 meters from the source.
• Airborne and fomite routes may contribute to BCV transmission.

## Graphical Abstract

**Figure:**
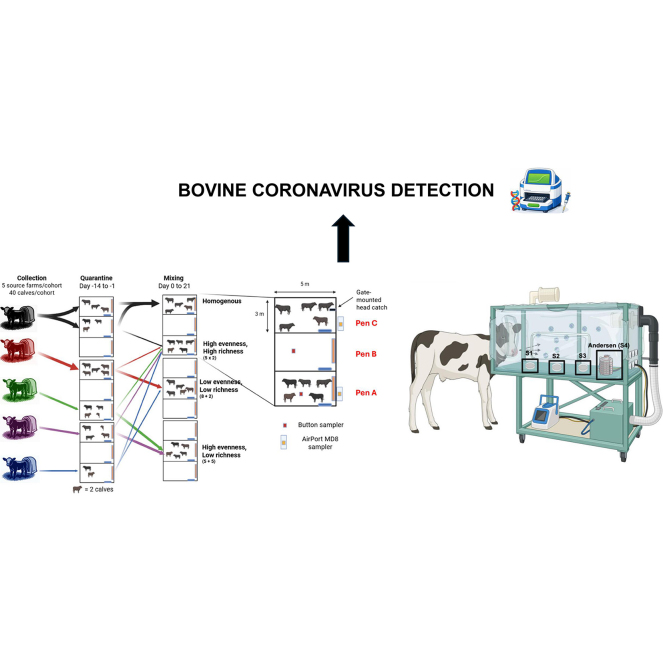
**Summary:**This study investigated the airborne and fomite transmission potential of bovine coronavirus (BCV) during a controlled commingling (mixing) experiment. Air, exhaled breath, and surface swab samples were collected from pens housing BCV-shedding calves and from an adjacent, unoccupied separator pen. Exhaled breath was sampled using a novel device capable of capturing particles at defined distances from the animal. Low levels of BCV RNA were detected in air samples from both occupied and unoccupied pens, at distances of at least 2.5 meters from the source. Surfaces within unoccupied pens were also contaminated with BCV RNA. Additionally, BCV RNA was detected in exhaled breath up to approximately 1.2 meters from the source. These findings indicate that BCV can be released into the environment through natural exhalation, supporting the potential role of airborne dispersal and fomite transmission during commingling events. Created with BioRender.com.

Bovine respiratory disease (**BRD**) has consistently ranked as the first or second most prevalent cause of morbidity and mortality in dairy calves for more than 2 decades (Short and Lombard, 2020). Bovine respiratory disease can be caused by numerous pathogens and often occurs as co-infection or sequential infections (Gaudino et al.*,* 2022). One of the most common viruses identified in recent diagnostic laboratory BRD submissions in Canada (28%; Buczinski et al.*,* 2024) and Belgium (38%; Pardon et al.*,* 2020) was bovine coronavirus (**BCV**). Although the clinical significance of BCV remains under discussion, it has been demonstrated that BCV vaccination reduces BRD treatment incidence in calves (Plummer et al., 2004) and improves average daily weight gain (Sandelin et al., 2020), making it a potentially relevant target for on-farm BRD management.

Bovine coronavirus is thought to be transmitted primarily via direct contact and fecal-oral routes (Saif, 2010), and its airborne transmission potential remains poorly understood. The recent pandemic of closely related betacoronavirus, SARS-CoV-2, highlighted the importance of airborne transmission in coronavirus epidemiology. Subsequently, several air sampling workflows were developed for human transmission studies (Cheng et al., 2020; Santarpia et al.*,* 2020); however, no published studies exist that evaluate bovine coronavirus presence in air within and surrounding calf housing facilities.

Therefore, this study aimed to evaluate the potential for airborne and fomite transmission of BCV by detecting viral RNA in the air and on environmental surfaces in group-housing calf facilities.

The study was conducted on weaned Angus × Holstein calves in association with a larger-scale study on BRD epidemiology (grant no. BB/Y006887/1) for which ethical approval was obtained (ASPA project PP6326528). Two cohorts (n = 40 animals per cohort) were evaluated as part of this work. Calves were obtained from 5 dairy farms for each cohort, and were between 8 and 10 wk old at enrollment. Calves were gradually weaned, with weaning completed by 8 wk of age according to their respective source farm (**SF**) protocols. Because 3 SF contributed to both cohorts, the study population originated from a total of 7 unique farms. Calves were transported to the research facility and quarantined within their SF groups for 12 to 14 d in solid-sided pens (Buitelaar High Health Units). Pens were 5 m deep and 3 m wide with smooth, solid double-walled plastic partitions from floor to ceiling. Pens had a single-span galvanized gate at the front (2.5 m from floor to ceiling) and solid wall at the back to a height of 1.22 m with a 1.0-m opening above to a total height of 2.22 m. An insulated box-profile roof spanned all 3 pens within one unit. There was a gap of 0.5 m between units. The layout of the pens was identical with a 30-L capacity wall-mounted water trough (DBL2, Sheep/Calf Drink Bowl, JFC Agri, Galway, Ireland) on the left of the pen looking in from the front and gate-mounted feed troughs (Gate Feeder, JFC Agri, Galway, Ireland). The pens were located outdoors with the front facing a westerly direction. Pens were bedded with straw which was supplemented daily. Calves were fed a TMR ad libitum, which was prepared on site.

Following quarantine, calves from each SF group were randomized within the strata of SF and sex, into one of 4 commingling groups: homogeneous (single-source pen); low evenness, low richness), which included 2 calves from one SF and 8 calves from a second SF; high evenness and low richness, which included 5 calves from one SF and 5 calves from a second SF; and high evenness and high richness, which included 2 calves from each of the 5 SF, which was pertinent to the aforementioned larger-scale BRD study. Pen order was randomized between groups to minimize potential pen location effect. Calves remained in their respective commingling groups for 21 d (Figure 1A).

**Figure 1:**
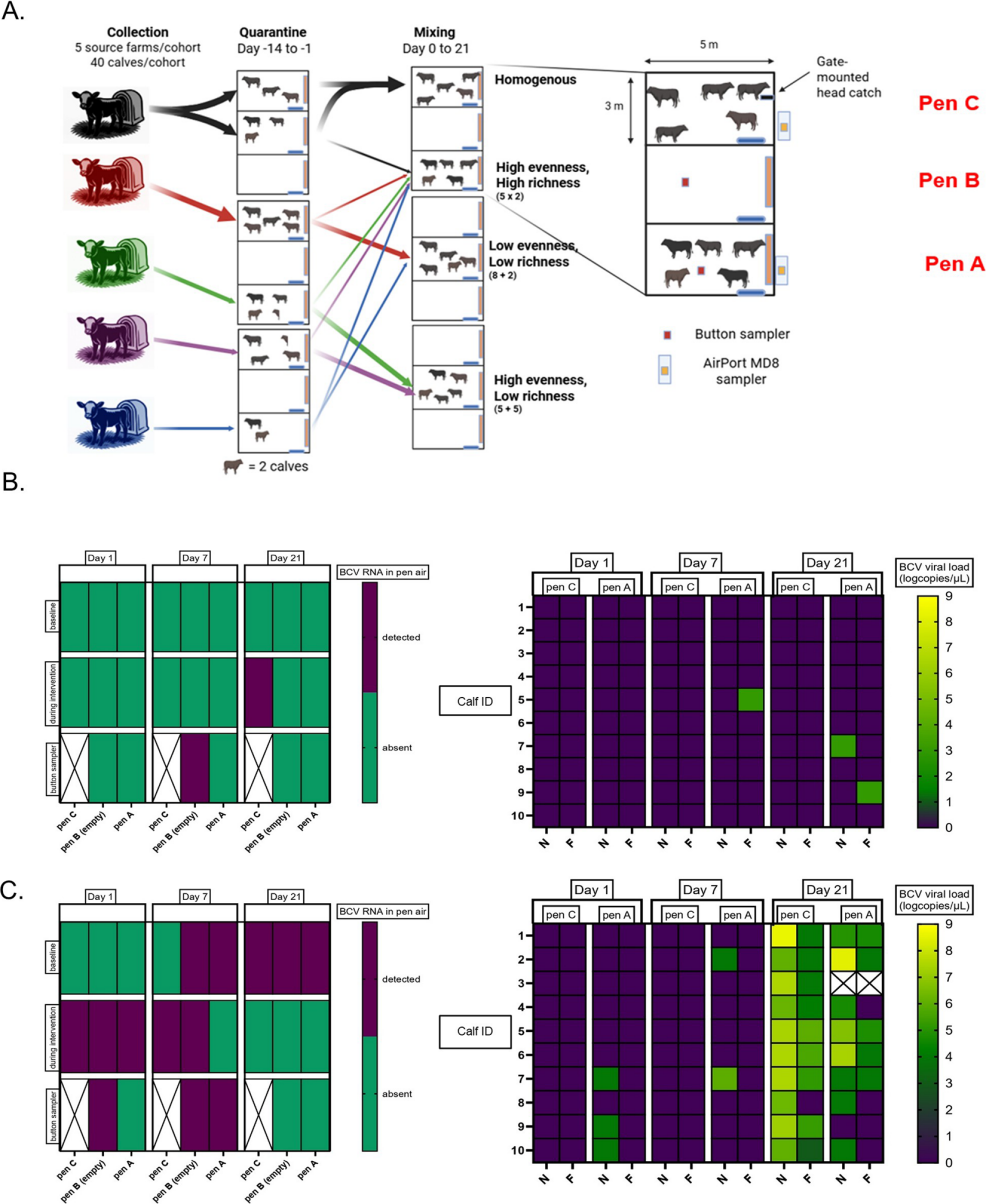
Bovine coronavirus (BCV) RNA detected in air samples. (A) Pen layout. Created with BioRender.com. (B) Cohort 1 and (C) cohort 2 pen air samples (left panels) and individual calf swabs (right panels). Air samples were collected in pens while calves were resting (baseline), during handling (intervention), and continuously (button sampler). N = nasopharyngeal shedding, F = fecal shedding. The limit of detection was 10^3^ copies/sample for environmental samples and 10^4^ copies/0.1-g swab for calf swabs.

Air and surface swab samples, as well as individual calf swabs were collected on d 1, 7, and 21 after mixing. Air sampling was synchronized with calf sampling to minimize the risk of interpen BCV transmission and to enable temporal correlation between environmental samples and calf BCV-shedding status. Pen entry was minimized throughout the study to reduce mechanical transmission risks. Straw bedding was applied manually without entering the pen, and calves were fed via gate-mounted troughs replenished using designated buckets for each pen. Disposable personal protective equipment (overalls, boot covers, and nitrile gloves) was worn and changed between pens during sampling. Boots were further disinfected by dipping and scrubbing in FAM30 disinfectant solution (Evans Vanodine) whenever moving between pens.

Air samples were collected from the mixed group (pen A), the empty separator pen (pen B), and the homogeneous group (pen C). Air samplers, SMD8 AirPort samplers (Sartorius AG), with a filtration rate of 50 L/min, were placed on a table at a height of 0.76 m and at a distance of 0.5 to 1 m from the front gates of the pens. Samplers were run for 30 min while calves were resting (the baseline sample) and again while calves were being handled in the occupied pen (during intervention sample). Button samplers (SKC Inc.) with a filtration rate of 4 L/min were placed on the ledge of a purlin at the height of 2.5 m and a horizontal distance of 1.25 m from the back wall of pens C and B and run continuously for 8 h on sampling days. Samples were collected onto sterile gelatin filters (Sartorius AG) and processed on the day of collection.

Surface samples were collected from all pens using standard flocked swabs with 2 mL of universal transport medium (**UTM**; Copan). Occupied pens were sampled twice: before and after calf handling. For each sample, an area of approximately 10 cm × 2 cm was swabbed 3 times, after which the tip was snapped off into the UTM vial. Sampling sites included the external surface of the water trough, back wall of the pen, and the front gate. Swabs were stored at −80°C until processing.

Deep nasal (**NS**) swab and fecal samples from each calf were collected on d 0, 7, and 21 of the study. The NS samples were collected using autoclaved 20-cm Jumbo Rayon-tipped swabs (Medline). The swab was inserted into the left nostril to a depth of approximately 15 cm and rotated 90° 3 times before removal. The tip was snapped off into 3 mL of DNA/RNA shield (Zymo Research) and stored at −20°C until further processing. Fecal samples were collected per rectum via digital stimulation or manual extraction into sterile pots and stored at −20°C until further processing.

Exhaled air was collected using a prototype IMADGENN (Isolator to Measure Aerosol and Droplet GENeratioN) particle isolator (PFI Systems Ltd., Milton Keynes, UK; Figure 2A). This purpose-built chamber (0.5 m wide × 1.6 m long × 1.7 m high) consists of soft-plastic walls with a front zip-operated panel for animal access. To ensure a low background aerosol level, the chamber is equipped with a HEPA-filtered air supply and extraction systems. The IMADGENN device operates with an airflow of 1.72 m^3^/min, providing approximately one air change every 45 s. Following sampling in a locking yoke, the calf's head was introduced through the zipped access point for 5 min, positioned so that the chin was resting on the chamber floor. Exhaled particles were detected by sedimentation on 25-mm gelatin filters (Sartorius AG) in sterile Petri dishes located approximately 40, 70, and 100 cm away from the chamber zipped access point. Simultaneously, a single-stage Andersen sampler (28.3 L/min) collected particles 1.2 m from the chamber access point. The isolator was disinfected by thorough spraying and wiping with 70% ethanol between animals.

**Figure 2:**
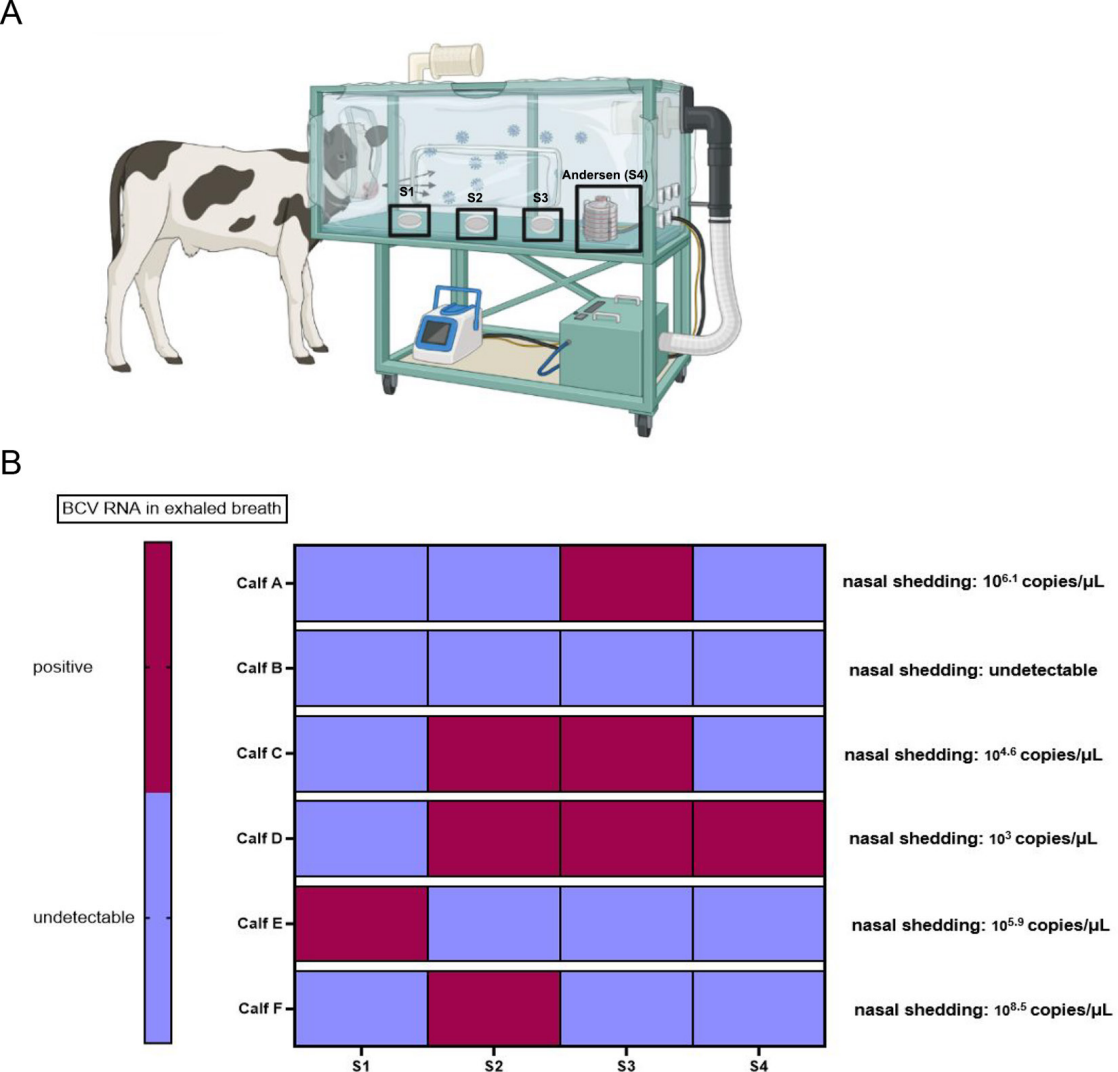
Bovine coronavirus (BCV) RNA was detected in naturally exhaled breath of shedding animals. (A) IMADGENN device setup. Particles were sedimented on gelatin filters at 40 (S1), 70 (S2), and 100 (S3) cm away from the calf head access point as the animal exhaled into the chamber. Aerosols were collected using a single-stage Andersen sampler (S4) positioned 1.2 m from the access point. Created with BioRender.com. (B) Bovine coronavirus RNA presence in exhaled particles at varying distances. Limit of detection was 10^3^ copies/sample. Presence or absence of nasal shedding was determined by qPCR of deep nasal swab taken on the same day.

Filters were aseptically transferred to a sterile Petri dish and dissolved overnight in 10 mL of Dulbecco's Modified Eagle Medium (Gibco), supplemented with 10% (vol/vol) heat-inactivated fetal bovine serum (Life Technologies) and 1% (vol/vol) 10,000 U/mL penicillin-streptomycin solution (ThermoFisher Scientific). Samples were stored at −80°C until further processing.

Total RNA from air filter and surface swab samples was extracted using a Quick-RNA Viral kit (Zymo Research), according to the manufacturer's instructions. Per extraction, 400 µL of sample was processed and RNA was eluted in 10 µL of RNase-free water. Media spiked with 10^10^ to 10^1^ copies of the BCV M gene RNA standard (Mebus strain bp 28841–29298, NCBI Taxonomy ID 11132) was used to determine the limit of detection of the assay, as previously described (Decaro et al.*,* 2008).

Total RNA from NS swabs was extracted with a Quick-DNA/RNA Miniprep kit (Zymo Research). One hundred milligrams of swab material was used per extraction. The swab was first homogenized using TissueLyzer II (Qiagen): 400 μL of kit lysis buffer was added to the weighed-out swab material in an Eppendorf tube containing 1 autoclaved 5-mm stainless steel ball (Sourcing MAP) and shaken for 3 min twice at 2.5 kHz. Afterward, the sample was centrifuged at room temperature for 1 min at 10,000 × *g*, the liquid was transferred to the extraction column and extraction proceeded according to manufacturer's protocol. RNA was eluted in 50 µL of RNase-free water.

Total RNA from fecal samples was extracted using a Quick-RNA Viral kit (Zymo Research) according to the manufacturer's instructions. Samples were preprocessed with the BioPrep MAP feces preparation kit (BioSellal, Dardilly, France) according to the manufacturer's instructions, with the exception of using viral RNA buffer (Zymo Research) rather than the supplied lysis buffer. Two grams of fecal material were processed per extraction, and RNA was eluted in 50 µL of RNase-free water.

Bovine coronavirus quantitative PCR (**qPCR**) primers and probe sequences had previously been validated by Decaro et al. (2008) and synthesized by Integrated DNA Technologies (Coralville, IA). Samples were amplified using Luna Universal Probe One-Step RT-qPCR Kit (New England Biolabs), according to the manufacturer's instructions. An annealing step (15 s at 50°C) was added to the standard protocol because it was found to increase reaction efficiency, and amplification was run for 35 cycles. Reactions were carried out on the Roche LC480 instrument (Roche Diagnostics) for NS swabs and fecal samples or the Biorad CFX96 instrument (BioRad) for surface swabs. A 10-fold increase in fluorescence relative to the baseline was used as positivity cut-off, and amplification curves were visually inspected to confirm amplification.

Low quantities of BCV RNA (∼10^3^ copies/sample) were recovered from air samples in both occupied pens and the empty separator pen (Figure 1B and 1C). Notably, BCV RNA was detected in the air of pen C on 3 occasions (cohort 1, d 21; cohort 2, d 1 and 7), despite the absence of detectable viral shedding by calves in that pen. However, concurrent shedding (either nasally or in the feces) was confirmed in at least 1 calf in the adjacent occupied pen (pen A), and in 2 of the 3 instances, BCV RNA was also detected in the empty separator pen. The minimum distance between the occupied pens (A and C), measured between the edge of pen walls, was 2.5 m.

Bovine coronavirus RNA (10^3^–10^4^ copies/sample) was detected in sedimented breath particles from 5 out of 6 sampled animals at varying distances (Figure 2B). All 5 breath-positive animals were confirmed as concurrent nasal shedders.

No BCV RNA was detected on pen surfaces in cohort 1 (occupied or empty), likely reflecting the low prevalence of shedding in this cohort (Table 1). In contrast, low amounts of BCV RNA (∼10^3^ copies/sample) were detected on surfaces in pens occupied by cohort 2, predominantly on the exterior of water troughs. On d 1, BCV RNA was detected on the water trough in a pen with BCV-positive calves and the adjacent empty pen. Although no BCV RNA was detected on surfaces on d 7, by d 21, coinciding with widespread nasal and fecal shedding, BCV RNA was detected on water trough surfaces in all pens, on the rear wall of one occupied pen, and on the front gate of the adjacent empty pen.

**Table 1:** Bovine coronavirus RNA detected on surfaces within occupied (A and C) and empty separator (B) pens during a controlled commingling event^1^

Pen	Cohort	Sampling day	BCV RNA			BCV+ animals^2^
			Trough	Gate	Wall	
C	1	1	ND	ND	ND	0/10
						
B	1	1	ND	ND	ND	(empty pen)
						
A	1	1	ND	ND	ND	0/10
						
C	1	7	ND	ND	ND	0/10
						
B	1	7	ND	ND	ND	(empty pen)
						
A	1	7	ND	ND	ND	1/10
						(10^4^ copies/μL)
						
C	1	21	ND	ND	ND	0/10
						
B	1	21	ND	ND	ND	(empty pen)
						
A	1	21	ND	ND	ND	2/10
						(10^3^ copies/μL)
						
C	2	1	ND	ND	ND	0/10
						
B	2	1	+	ND	ND	(empty pen)
			(10^3^ copies/μL)			
						
A	2	1	+	ND	ND	3/10
			(10^3^ copies/μL)			(10^4^ copies/μL)
						
C	2	7	ND	ND	ND	0/10
						
B	2	7	ND	ND	ND	(empty pen)
						
A	2	7	ND	ND	ND	2/10
						(10^4^–10^6^ copies/μL)
						
C	2	21	+	ND	ND	10/10
			(10^3^ copies/μL)			(10^4^–10^8.6^ copies/μL)
						
B	2	21	+	+	ND	(empty pen)
			(10^3^ copies/μL)			
						
A	2	21	+	ND	+	8/9
			(10^3^ copies/μL)		(10^3^ copies/μL)	(10^4^–10^8.5^ copies/μL)

1 Limit of detection was 10^3^ copies/sample for environmental swabs and 10^4^ copies/0.1-g swab for calf samples. ND = not detected.

2 BCV+ refers to calves in the pen shedding detectable BCV either nasally or in the feces on the day of sampling.

This study demonstrated that BCV RNA is present in exhaled breath of BCV-shedding calves, as well as in surrounding air and on environmental surfaces. To our knowledge, airborne detection of BCV has not been reported previously; however, comparable findings have been described for other coronaviruses, such as MERS-Cov1 RNA fragments found in camel barn air samples (Azhar et al.*,* 2014). Although the detection of viral RNA does not equal airborne infectivity, the recovery of BCV RNA from air of both occupied and adjacent pens suggests that infectious material may become airborne under typical husbandry conditions. Isolation of virions from air samples is technically challenging due to low viral loads and potential physical damage to viral particles during the sampling process (Silva et al., 2022). Our finding challenges the prevailing assumption that airborne transmission of BCV is of limited importance (Stokstad et al., 2020), and implies that current biosecurity strategies focused solely on limiting direct contact transmission may be insufficient.

Beyond airborne virus detection, we identified widespread environmental contamination consistent with reports of coronavirus fomite transmission (Onakpoya et al.*,* 2022). Notably, viral RNA was also detected on the water trough surface of an empty separator pen positioned between BCV-shedding groups. We hypothesize that this contamination occurred via sedimentation of airborne particles dispersed from adjacent pens, a theory supported by the detection of BCV RNA in air samples collected from this unoccupied space. It should be noted that this pen had been occupied by calves during the quarantine period (until approximately 24 h prior to sampling d 1); however, none were shedding detectable levels of BCV either nasally or in the feces.

Environmental factors strongly influence the behavior and persistence of virus-laden droplets and aerosols. Although droplet size largely determines travel distance, temperature and relative humidity also play key roles in airborne transmissibility (Drossinos et al.*,* 2021). This study was conducted in the United Kingdom during autumn and winter when low temperatures (<15°C) and high humidity (>60%) are commonplace. Although lower temperatures generally increase viral stability, high humidity tends to favor faster droplet settling, thereby increasing surface deposition and decreasing longer-range particle transmission (Božič and Kanduč, 2021). These factors might partly explain more extensive surface deposition at certain timepoints. Incorporating environmental monitoring in future studies could help clarify how seasonal or microclimatic variation affects BCV aerosol persistence and transmission potential.

Finally, we successfully analyzed exhaled breath samples using a controlled airflow chamber and detected BCV RNA up to 1.2 m from the calf. Such technology can improve our understanding of dispersal patterns of BCV, as well as provide a methodology framework for other BRD pathogens. Isolation of viral RNA directly from breath-borne particles has been attempted previously for bovine respiratory syncytial virus (Santos-Rivera et al., 2022) and foot-and-mouth disease virus (Christensen et al.*,* 2011), but not BCV. Furthermore, if required, the sampling method developed in this study allows for isolation of complete viral particles for subsequent cell-infectivity assays.

In summary, our findings suggest that BCV RNA can be released into, and disperse through, air via exhalation, suggesting that airborne and fomite routes could contribute to transmission risk in group housing. Although definitive demonstration of airborne transmission requires additional work, this study provides a methodological framework for detecting and characterizing airborne pathogens in cattle. The approach described here can be readily adapted to other BRD agents, supporting future efforts to quantify transmission risks and refine biosecurity strategies for the cattle industry.
